# The genetic control of growth rate: a systems biology study in yeast

**DOI:** 10.1186/1752-0509-6-4

**Published:** 2012-01-13

**Authors:** Pınar Pir, Alex Gutteridge, Jian Wu, Bharat Rash, Douglas B Kell, Nianshu Zhang, Stephen G Oliver

**Affiliations:** 1 Cambridge Systems Biology Centre and Department of Biochemistry, University of Cambridge, Sanger Building, 80 Tennis Court Road, Cambridge CB2 1GA, UK; 2 Faculty of Life Sciences, The University of Manchester, Michael Smith Building, Oxford Road, Manchester M13 9PT, UK; 3 Manchester Interdisciplinary Biocentre and School of Chemistry, The University of Manchester, 131 Princess Street, Manchester M1 7DN, UK

## Abstract

**Background:**

Control of growth rate is mediated by tight regulation mechanisms in all free-living organisms since long-term survival depends on adaptation to diverse environmental conditions. The yeast, *Saccharomyces cerevisiae*, when growing under nutrient-limited conditions, controls its growth rate via both nutrient-specific and nutrient-independent gene sets. At slow growth rates, at least, it has been found that the expression of the genes that exert significant control over growth rate (high flux control or HFC genes) is not necessarily regulated by growth rate itself. It has not been determined whether the set of HFC genes is the same at all growth rates or whether it is the same in conditions of nutrient limitation or excess.

**Results:**

HFC genes were identified in competition experiments in which a population of hemizygous diploid yeast deletants were grown at, or close to, the maximum specific growth rate in either nutrient-limiting or nutrient-sufficient conditions. A hemizygous mutant is one in which one of any pair of homologous genes is deleted in a diploid, These HFC genes divided into two classes: a haploinsufficient (HI) set, where the hemizygous mutants grow slower than the wild type, and a haploproficient (HP) set, which comprises hemizygotes that grow faster than the wild type. The HI set was found to be enriched for genes involved in the processes of gene expression, while the HP set was enriched for genes concerned with the cell cycle and genome integrity.

**Conclusion:**

A subset of growth-regulated genes have HFC characteristics when grown in conditions where there are few, or no, external constraints on the rate of growth that cells may attain. This subset is enriched for genes that participate in the processes of gene expression, itself (*i.e. *transcription and translation). The fact that haploproficiency is exhibited by mutants grown at the previously determined maximum rate implies that the control of growth rate in this simple eukaryote represents a trade-off between the selective advantages of rapid growth and the need to maintain the integrity of the genome.

## Background

Appropriate control of the rate of cell growth is central to the long-term survival of species, particularly microorganisms. A fast growth rate is a competitive advantage when environmental conditions are favourable, while slow growth or even quiescence may allow survival under stress conditions such as rapid changes in the physicochemical environment, starvation, or exposure to toxins. Thus most organisms have evolved stringent controls over the rate of cell growth, and any disruption of these controls has severe consequences; an obvious example being the development of cancer as a result of uncontrolled cell division.

Our genome-scale approach to the investigation of growth rate control in the model eukaryote cell, *S. cerevisiae*, is based on the concepts of Metabolic Control Analysis (MCA; [1]). MCA defines the extent of control exerted over the flux through a pathway by its components. In our context, flux is the growth rate of cells, pathway components are the gene products, and control coefficients are the ratios of the fractional changes in growth rate to the fractional changes in the concentrations of gene products. Thus the term "control" has a special meaning in the context of MCA. Those cell components that exert control over flux are not necessarily regulatory molecules. Rather, they exert control because the flux through the pathway is *sensitive *to changes in the concentration (or activity) of the component. In this paper, we shall routinely use the term 'growth rate', rather than 'flux', since we have measured differences in growth rate consequent on reducing the copy number of individual genes from two to one in diploid yeast.

As part of our overall approach, we have designed experiments in two categories to elucidate the control coefficients of gene products for growth rate. In Category 1 experiments, we measured the change in the concentration of gene products as a response to a change in flux ([2];[3]). We report no new Category 1 experiments in the present study, but we shall refer to our earlier experiments in this Category. In Category 2 experiments, the concentration of gene products is altered and any impact on growth rate measured [4]; all new data reported in this paper relates to Category 2 experiments.

In our earlier Category 1 experiments, we grew a reference yeast strain in carbon-, nitrogen-, phosphorus-, or sulphur-limited conditions in chemostat culture at dilution rates, D = 0.07, 0.1 and 0.2 h^-1 ^(equivalent to doubling times ~ 10, 7, and 3.5 h, respectively), and collected samples at each of the different steady states. We analysed our samples for changes in the levels of mRNAs, proteins, and metabolites with respect to dilution rate and identified a set of growth-rate-regulated (GR) genes; *i.e. *a set of genes that significantly changed their expression levels in response to the change in growth rate, irrespective of the specific nutrient whose rate of supply determined the rate of growth [2]. There is also a nutrient-regulated (NR) gene set, whose expression levels change according to the nutrient whose rate of supply is determining the growth rate; again, we have reported on these previously [3]. The level of expression of NR genes may vary in either a growth-rate-dependent or growth-rate-independent manner.

In the set of Category 2 experiments that we reported previously, we established a growth-rate competition between yeast deletion mutants (each hemizygous for just one of the organisms 5,800 protein-encoding genes [5]) under carbon, nitrogen, or phosphorus limitation in chemostats at D = 0.1 h^-1 ^[4]. We classified all the mutants according to the sign of their relative growth rate, the mutants with negative relative growth rate *(i.e. *whose proportion in the population fell significantly overtime) were classified as haploinsufficient (HI) and the mutants with positive relative growth rate *(i.e. *whose proportion in the population significantly increased with time) were classified as haploproficient (HP). The set of haploinsufficient and haploproficient genes together form the set of high flux control (HFC) genes, *i.e. *those genes for which a reduction of their copy number in diploid cells from 2 to 1 results in a significant change in growth rate (flux).

Our earlier results showed that, under all three nutrient limitations and at a dilution rate of 0.1 h^-1^, there was little overlap between HFC genes from Category 2 experiments and the GR genes defined in Category 1 experiments; *i.e. *the genes regulated by growth rate are not, themselves, regulators of growth. At the time [4], we noted that this result might, in the jargon of Systems Biology, represent a 'design rule' for the eukaryotic cell. However, since cells are the products of evolution rather than design, such rules may change according to the selection pressures to which an organism is exposed. Thus, while this rule about HFC genes holds for nutrient-limited environments, further studies are required to determine whether it has any greater generality. This paper reports these additional studies.

Since our previous set of Category 2 experiments was carried out at a low growth rate and under nutrient limitation, we decided to determine whether the lack of overlap between HFC and GR gene sets still held at high growth rates or under nutrient-unconstrained conditions. Chemostats tend to be unstable at dilution rates close to the cells' maximum specific growth rate (μ_max_) and so, to establish a steady state at μ_max_, we used turbidostats to allow us to monitor competition between the pool of hemizygous yeast deletants growing in a complex synthetic medium (FPM [6]; see Methods section for modifications). Turbidostats are continuous cultures in which the cells, rather than the experimenter, control the growth rate [7] since the nutrient supply is determined by the biomass concentration in the growth vessel. Accordingly, we established a turbidostat in which the biomass concentration was held at a value equivalent to that of a mid-exponential phase batch culture. The turbidostat is unconstrained by the supply of nutrients and so equilibrates at the maximum specific growth rate of the yeast strain used. However, if the culture is a pool of hemizygous mutants of *S. cerevisiae*, and if the different mutants in the pool can have different maximum growth rates, those mutants that can achieve a μ_max _greater than the population average will increase in the population over time, while those with a μ_max _less than the population average will decrease in the population. (Those readers unfamiliar with chemostats and turbidostats will find a fuller explanation in Additional File 1, Additional Text; also see reviews by Bull [8] and Pirt [7].)

Turbidostats, just like chemostats, represent a sensitive way of identifying haploinsufficient and haploproficient phenotypes, with the difference that (in a turbidostat) haploproficient mutants must be capable of growing at a rate greater than the previously recorded μ_max_. Thus, in this study, we have searched for sets of genes that exhibit either haploinsufficient or haploproficient phenotypes (these are both sub-sets of the class of HFC genes) in rapidly growing yeast cultures, either in nutrient-limited chemostats operated at D-values close to μ_max _or in the nutrient-unconstrained conditions of turbidostat culture. Further, we investigated subsets of HFC genes that have closely related cellular functions in order to elucidate the gene and protein features that mediate their high degree of control over growth rate. Finally, we propose an extended approach to growth rate (or flux) control, without limiting its definition to enzymes or haploinsufficiency.

## Results

### Genes showing high growth-rate control during rapid growth in nutrient-unconstrained conditions

After monitoring the competitive growth of the mutants in the turbidostat for about 30 generations, we calculated the relative change in the log.-transformed abundance of mutants in the pool with respect to time:

FCC'=d(lnX)dt(see Methods section for derivation).

The mutants were then classified according to the sign of their relative growth rate. 796 mutants were identified with a positive slope (FDR < 0.05). These grew faster than the population average and are defined as haploproficient. 1932 mutants grew slower than the population average (FDR < 0.05) and are defined as haploinsufficient. In total, 2728 HFC genes were found for which the changes in the relative concentrations of their hemizygotes were not significant (see Additional File 1, Additional Text; Additional File 2, Figure S1; Additional File 3, Table S1).

We examined whether particular functional classes of genes were enriched among the HI and HP sets using GO terms [9] and logistic regression [10] (Table 1; see Additional File 4, Table S2 for complete results for GO terms). Genes associated with the processes of transcription and translation, *e.g. *genes that encode cytosolic ribosomal proteins and the components of the RNA polymerase I, II, and III complexes were enriched in the HI gene set at a high level of significance *(P *< 10^-5^). Apart from the functional categories, it was also noted that genes whose products are members of macromolecular complexes were highly enriched in the HI set (*P *< 10^-9^). This was true even if genes encoding ribosomal proteins were excluded from the HI set.

**Table 1:** GO terms enriched among HFC control (see Additional File 4, Table S2 for complete list of GO terms)

	ENRICHED HAPLOINSUFFICIENT SET			
**GO ID**	**GO term**	**Ontology**	**Number of genes**	**FDR**

GO:0005840	ribosome	CC	287	3.55E-018

GO:0030880	RNA polymerase complex	CC	31	6.83E-016

GO:0055029	nuclear DNA-directed RNA polymerase complex DNA-directed RNA polymerase II, core	CC	31	6.83E-016

GO:0005665	DNA-directed RNA polymerase II, core complex	CC	12	3.51E-014

GO:0016591	DNA-directed RNA polymerase ll, holoenzyme	CC	70	4.70E-012

GO:0032991	Macromolecular complex	CC	1538	4.22E-010

GO:0005736	DNA-directed RNA polymerase I complex	CC	14	4.23E-006

GO:0005832	chaperonin-containing T-complex	CC	11	1.57E-005

GO:0005666	DNA-directed RNA polymerase III complex	CC	17	2.18E-005

GO:0032200	telomere organization and biogenesis	BP	260	4.25E-005

GO:0042254	ribosome biogenesis and assembly	BP	334	9.79E-005

GO:0000119	mediator complex	CC	21	0.000265

GO:0005838	proteasome regulatory particle	CC	20	0.014216

GO:0030528	transcription regulator activity	MF	335	0.014216

GO:0004175	endopeptidase activity	MF	77	0.027946

	**ENRICHED HAPLOPROFICIENT SET**			

**GO ID**	**GO term**	**Ontology**	**Number of genes**	**FDR**

GO:0007049	cell cycle	BP	552	0.002534

GO:0051320	S phase	BP	20	0.003251

GO:0006260	DNA replication	BP	151	0.010963

GO:0015932	nucleobase, nucleoside, nucleotide and nucleic acid transmembrane transporter activity	MF	13	0.017113

GO:0000082	G1/S transition of mitotic cell cycle	BP	52	0.023716

GO:0005669	transcription factor TFIID complex	CC	14	0.034692

GO:0005685	snRNP U1	CC	15	0.035741

GO:0051052	regulation of DNA metabolic process	BP	41	0.043266

GO:0016020	membrane	CC	1592	0.044202

GO:0005991	trehalose metabolic process	BP	10	0.050603

Analysis of the smaller set of HP genes from the turbidostat selection showed that the over-represented functional categories included the cell cycle (*e.g. *spindle checkpoint genes) and macromolecule storage (*e.g. *genes encoding proteins involved in trehalose metabolism). The fraction of HFC genes in various organelles, selected protein complexes and cellular processes is shown in Additional File 5, Figure S2.

### HFC at rapid growth in comparison to nutrient-limited chemostats at low growth rates

**Figure 1:**
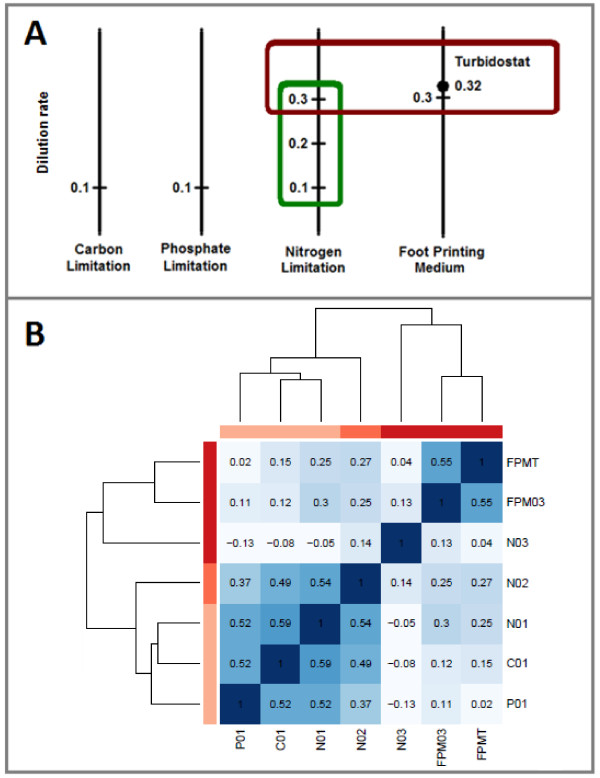
**Experimental design and correlation of growth profiles in different conditions**. **A**: Competition in carbon-, nitrogen- or phosphorus-limited (F1) chemostats operating at 0.1 h^-1 ^[4] were initially compared to competition in turbidostat cultures using complete synthetic medium (FPM, [6]; this work). Nitrogen-limited chemostat experiments at 0.2 h^-1 ^and 0.3 h^-1 ^(this work) and FPM fed chemostat at 0.3 h^-1 ^(this work) were designed to investigate the differences between the competition in nutrient-limited chemostats operated at low growth rates to competition in the high growth-rate nutrient-sufficient conditions of the turbidostat. B: Correlation matrix based on the Spearman correlation coefficient of the relative growth rates of each hemizygote obtained from each experiment. The dendrograms are calculated using the Euclidean distance between rows (experiments) and hierarchical clustering.

Results from the present study demonstrate that the functional distribution of HFC genes detected by competitions in turbidostats is very different from the HFC genes detected by our earlier Category 2 experiments [4] that involved competitions in nutrient-limited chemostats. However, it is not clear if this difference is a result of nutrient availability or growth rate, since the experiments by Delneri and co-workers were performed at a relatively slow growth rate (D = 0.1 h^-1^), while cells in a turbidostat grow at their maximum rate. To address this question, we carried out additional competition experiments aimed at distinguishing between the impact of fast growth and that of nutrient sufficiency (See Figure 1A for the experimental design).

First, a yeast culture in synthetic complete medium (FPM) was grown in chemostat mode at D = 0.3 h^-1^. This means the same growth conditions were applied as in the turbidostat except that the cells were growing at a fixed dilution rate close to the maximum specific growth rate. The dilution rate in the turbidostats, and hence the maximum growth rate of the pool, stabilised at 0.32 h^-1^. However, 0.3 h^-1 ^was used in the chemostat, because operating at 0.32 h^-1 ^would be at the limit of the stability for a chemostat culture of the pool of mutants. We found that the set of HFC genes detected under these conditions was very similar to the set detected in turbidostat competitions (Additional File 3, Table S1). Thus, similar sets of HFC genes were found at similar growth rates despite the fact that one culture was nutrient-limited (the chemostat with FPM is leucine-limited), while the other was nutrient sufficient (turbidostat).

Next, we carried out competitions in ammonium-limited chemostats at higher dilution rates (D = 0.2 h^-1 ^and 0.3 h^-1^). We will refer to these two conditions as N02 and N03, respectively, with a nitrogen-limited chemostat at D = 0.1 h^-1 ^being termed N01; correspondingly cultures grown in FPM are termed FPMT, if run in turbidostatic mode, and FPM03, if run as a chemostat at D = 0.3 h^-1^. First of all, the results demonstrated that the HFC genes detected under nitrogen limitation vary considerably at different dilution rates. The correlation of FCC's (Figure 1B) from N01 and N02 is 0.54, indicating that most mutants behave similarly under the two conditions, however when dilution rate is increased further to 0.3, the similarity disappears. Competitions with different nutrient limitations (C01, N01 and P01) are highly correlated to each other at a slow growth rate, and cluster together on the dendrogram. The fast-growth rate experiments cluster together (FPMT, FPM03 and N03) and are highly correlated to each other. Hence the major difference between the competitions is the rate of growth achieved, rather than the nutrients being in plentiful or limiting supply.

### Genetic control of growth rate is qualitatively, but not quantitatively, correlated with, growth rate regulation of gene expression

**Figure 2:**
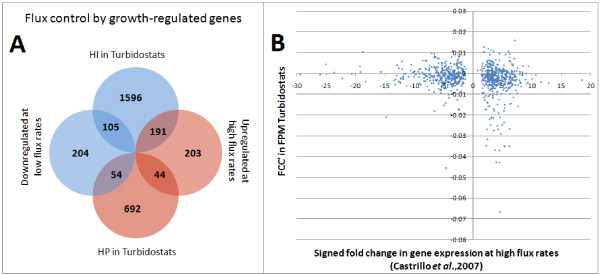
**Comparison of FCC's of genes in turbidostat competition to regulation by growth rate**. Only the genes significantly regulated by growth rate are shown. A. The overlap between HFC genes identified by selection in turbidostats (this work) and up-regulation at high dilution rates [2] is significant. B. No significant correlation can be found between a gene's expression being regulated by growth rate and its FCC' value in turbidostat culture.

Previously, we have shown that genes involved in the processes of gene expression are highly up-regulated at high growth rates (GR genes from Category 1 experiments, [2]). These significantly up-regulated genes show extensive identity to genes identified as HI at high growth rates (hypergeometric p < 10^-5^) and are highly underrepresented in the set of HP genes (hypergeometric p < 10^-32^) (Figure 2A). However, while the degree of overlap between the GR and HI gene sets is high, we find that (in quantitative terms) the fold change of up-regulation in gene expression is only weakly correlated to the FCC values (rho = -0.15) (Figure 2B). This demonstrates that, although growth-regulated genes are, themselves, controllers of growth rate at the fastest growth rates, there is no simple relationship between the change in growth rate due to haploinsufficiency and the fold change in transcript level or protein concentration of those same HI genes at different growth rates.

It should be noted that, although Category 1 experiments showed the transcript levels of genes encoding components of the mitochondrial ribosome to be up-regulated as a function of growth rate in chemostats, the competition experiments in turbidostats did not indicate that any growth-rate control was exerted by these components. Although mitochondrial activity has long been known to be an important factor that affects the growth rate in yeast (*e.g. *respiratory-deficient *petite *mutants are slow-growing [11]), our competition experiments do not suggest that genes encoding mitochondrial proteins exert any significant growth-rate control. Expression of the mitochondrial ribosomal proteins were up-regulated in C-limited chemostats at high dilution rates [2], indicating high levels of respiration were taking place at higher dilution rates under carbon limitation. The lack of growth-rate control by mitochondria in our experiments could be an outcome of the high glucose concentration in both the turbidostat and the nitrogen-limited chemostat at D = 0.3 h^-1^, where excess glucose would repress respiration and promote fermentation.

### Range of flux control varies among genes within the same functional category

**Figure 3:**
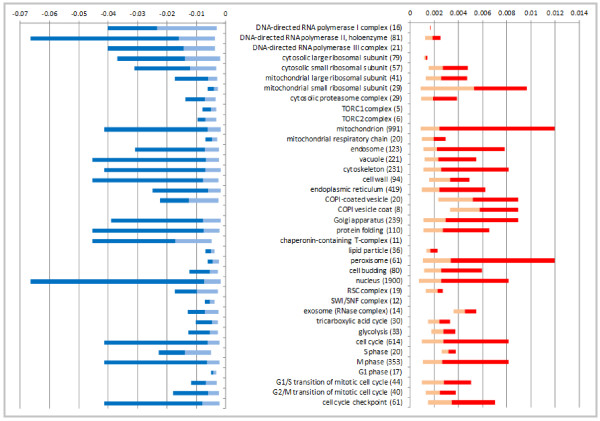
**Range of FCC' values for HFC genes belonging to the categories shown in Additional Figure 2**. On the left, light blue bars show the range of FCC's of significantly HI genes with smaller magnitude than the average for the category, dark blue bars shown the range of FCC's of significantly HI genes with larger magnitude than the average for the category (the junction of the light and dark blue bars is the average FCC' of the category). On the right, light red bars show the range of FCC's of significantly HP genes with smaller magnitude than the average of the category, dark red bars show the range of FCC's of significantly HP genes with larger magnitude than the average of the complex (the junction of the light and dark red bars is the average FCC' of the complex). It should be noted that ranges of FCC' values for HI genes are larger than those for HP genes. The numbers in parentheses give the number of genes in the category with data from competition experiments.

The control of growth rate by genes involved in large cellular components can be of different strength or even of negative directions. For example genes encoding the proteins located in nucleus or in mitochondrion can have a large range of FCC's, spanning from positive FCC's with large magnitude to negative FCC's with large magnitude, including the 'weak range', where FCC's are very close to zero but still are significant (Figure 3). However, even protein complexes specialized on a single function, like ribosomes, can have components with a large range of FCC's.

Most protein complexes are essential for viability, though not every component of a given complex is essential. By analogy, it can be hypothesized that most protein complexes exert growth-rate control when cells are growing rapidly, though not every gene that encodes a component of a particular has a significant flux control coefficient (FCC'). We have selected a subset of protein complexes and cellular processes to exemplify the range of FCC' values in turbidostats (Figure 3; Additional File 5, Figure S2). A highly haploinsufficient small protein complex like RNA polymerase I, can include a few HP genes with a small range of FCC's, while the genes encoding the rest of its subunits have a large range of negative growth-rate control coefficients. Moreover, the average FCC' values for members of different multiprotein complexes was found to vary in turbidostat competitions. For instance, genes encoding sub-units of the COPI vesicle coat and the exosome have high average positive FCC' values, while those encoding components of the RNA polymerase I complex and the chaperonin-containing T-complex have high average negative FCC's.

### Genes with functions involved in the cell cycle and the maintenance of genome integrity are associated with haploproficiency

Genes involved in the cell cycle that determine the G1/S phase transition and progression through S phase were particularly enriched in our set of haploproficient genes. A subset of cell cycle related genes are expressed periodically as the cell cycle progresses [12] and regulated post-transcriptionally by a sequence of events [13] including 'just-in-time assembly' [14]. A reduction in the copy number of some of these genes in the results in growth rate above μ_max _in turbidostats (Additional File 3, Table S1). This indicates that a reduction of controls that delay the cell cycle under normal conditions can, in fact, allow the cycle proceed more quickly in these HP hemizygotes.

**Figure 4:**
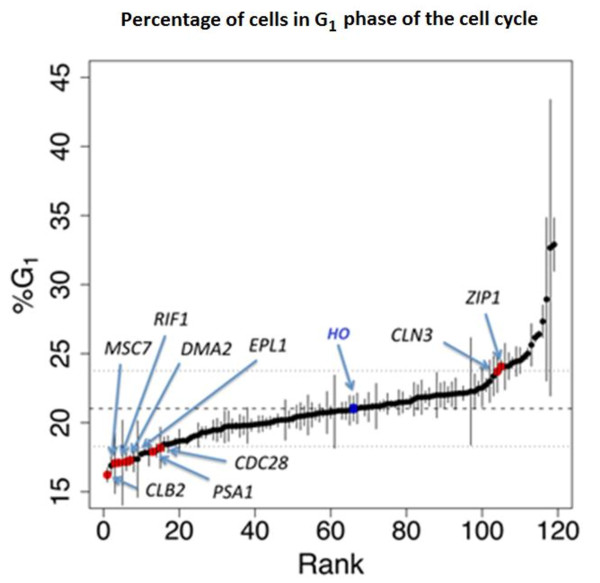
**The effect on of hemizygous deletions on progress through the cell cycle**. The percentage of cells in G1 phase of the cell cycle during exponential growth is shown on the vertical axis for 120 heterozygous deletion mutants. The mean length of G1 as a percentage of the total length of the cell cycle is shown for all strains (bars show ± 1 standard deviation from the mean). The *HO/ho *mutant strain used as a control is shown in blue. Strains showing changes in the cell cycle > 1 sd and significant haploproficiency (P < 0.001) in one or more of the D > = 0.3 h^-1 ^experiments are highlighted in red, along with cyclin gene *CLB2*, which does not show haploproficiency, but shows a strong cell cycle phenotype.

Having observed that cell-cycle functions were associated with haploproficiency at higher growth rates, we measured (for 120 hemizygotes), the proportional distribution of cells in different phases of the cell cycle (Figure 4). Previously, wild-type cells growing in chemostats at different growth rates were reported to have the length of their unbudded G1 or G0 phase negatively correlated with their growth rate [15]. We compared the G1-phase enrichment of the 120 mutants to their flux control coefficients in turbidostat culture, and found no significant correlation (rho = -0.14, p-value = 0.14). This demonstrated that the haploproficiency of these mutants is not an immediate result of a shortened G1-phase.

Amongst those mutants that showed a large change in their cycle maps were deletants of *CDC28 *and two cyclin genes, *CLB2 *and *CLN3*, whose products promote the G2/M and G1/S transitions, respectively [16,17]. Both *CLN3 *and *CDC28 *were HP at high growth rates, suggesting a link between altered progress through the cycle and faster growth. We have selected a subset of 9 genes that control the G_2_/M transition to investigate their effect on progress of cell cycle and phenotype in tetraploid deletion mutants (Alcasabas and co-workers, submitted). Other genes showing both haploproficiency and an altered cell cycle included *DMA2*, whose human ortholog *RNF8 *regulates the G_2_/M DNA damage checkpoint [18], and *EPL1*, a component of the NuA4 complex, which has also been linked to DNA repair [19]. We will now discuss in more detail the set of haploproficient genes involved in the maintenance of genome integrity.

Genome integrity related functions are tightly linked to cell cycle as replication of genome requires timely functioning of various protein and protein complexes regulated by cell cycle. 207 of the 384 genes annotated to the GO term 'chromosome organisation' are HFC genes. Many of these genes, as well as others, are directly involved in the maintenance of genome integrity and also show a haploproficient phenotype. These genes and their functions (some 42 of them are listed in Additional File 6, Table S3). It is clear from the results of the turbidostat competition experiments that yeast is capable of growing at faster than maximum specific growth rate of the wild-type strain. However, the imperative of correctly replicating and segregating its chromosomes, repairing DNA damage, and generally maintaining its chromosome integrity has imposed restraints on the absolute rate of growth that can be sustained over evolutionary time.

### Growth-Rate Control is a Complex Function of Protein Activity

The relationship between gene copy number and protein levels is an important factor that must be taken into consideration when investigating growth-rate control. Torres and co-workers [20] have reported that aneuploid yeast cells overexpress the genes on the extra chromosomes (93% of the genes on the extra chromosomes were found to be overexpressed by at least a factor of 1.3-fold compared to the wild-type haploid parent). Recently, it has also been reported that, for most genes tested, the average level of their protein product is proportional to gene copy number, which indicates that dosage compensation is a rare event [21].

Springer and co-workers [21] investigated 643 genes and 123 of them were found to have significant levels of dosage compensation when one copy was deleted from the genome, while 18 genes reduced their expression to less than half of the wild-type level, when one copy is deleted from the genome. We reasoned that more HI genes should be found in the set of genes that exhibit no dosage compensation and more HP or non-HFC genes in the set of genes that exhibit some dosage compensation. This is because a lack of dosage compensation is more likely to result in inadequate protein concentrations in the cell, and thus result in a reduction of fitness. Genes with dosage compensation would be expected to have less growth-rate control as the concentrations of their protein products in a hemizygote will be closer to those in the wild type. Furthermore, haploproficiency can be expected if dosage compensation leads to product levels that exceed those of the wild type. We have compared the dosage compensation values [21] (log2 transformed fold changes, 0 indicates no dosage compensation, 1 indicates dosage compensation that matches the wild-type level of the protein) to FCC' values from turbidostat competitions and the overlap of HFC genes to genes with or without significant dosage compensation.

**Figure 5:**
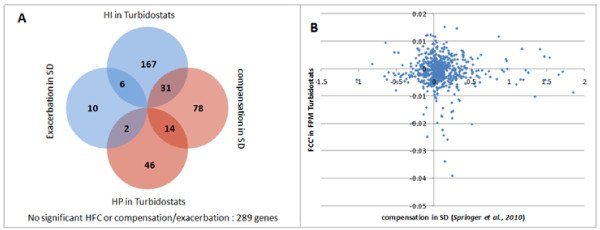
**Comparison of FCC's of genes in turbidostat competition to dosage compensation**. Only the genes studied by Springer and co-workers [42] are shown. A. The overlap between HFC genes in turbidostats and genes with compensation or exacerbation is not significant. B. No significant correlation (Spearman rho = 0.016, p-value = 0.69) can be found between dosage compensation and FCC'. (Note: Correlation of FCC' to dosage compensation reported by Torres and co-workers' [20] is rho = 0.01, p = 0.83).

The genome-wide frequency of HI genes identified in our turbidostats is 34%, while the frequency of HI genes in the sets of genes with and without dosage compensation is 25% and 33% (31/123 and 167/502), respectively (Figure 5A), indicating that HI genes are only slightly underrepresented in the set of genes with dosage compensation (hypergeometric p-value = 0.0246), rather than being depleted. Thus, there is no significant correlation (Figure 5B) between genes showing dosage compensation and those with an HFC phenotype in turbidostats or in any of the other conditions that we investigated. This indicates that there is no simple relationship between either a protein's level orthe dosage compensation of its cognate gene and the HFC characteristics of that gene.

It was further reported by Springer and co-workers [21] that dosage compensation remains the same for a protein across different growth conditions. If dosage compensation (or the lack of it) was a simple indication of high growth-rate control, then we would expect to find a similar set of HFC genes in different conditions, and flux control coefficients to be (negatively) correlated to dosage compensation, both of which we failed to find. Hence, it should be realised that the flux control exerted by a protein is a function of its biological activity (which is probably context dependent), rather than simply on its molecular abundance. None of the high-throughput studies (including our own) measures biological activity, and so it might be anticipated that FCC' values are a complex function of protein abundance.

Few other factors were shown to link protein abundance to growth-rate control. For example, Sopko and co-workers [22] found that *ca. *15% of the genes in yeast are toxic when overexpressed; that is, their overexpression reduced the growth rate. This set of toxic genes was enriched in cell cycle genes, particularly in genes that are expressed periodically. This finding supports the hypothesis that a protein can be toxic even at its wild-type levels and thus the expression of its cognate gene has to be carefully controlled. Such toxicity may increase as the protein abundance increases. It follows that a reduction in the concentration of a protein that acts as an inhibitor of growth at its wild-type concentration can have a positive impact on growth rate. Yoshikawa and co-workers [23] reported that deletion and overexpression mutants of more than 400 genes have growth defects. (Note that very few deletion or over-expression mutants that have a haploproficient phenotype were found -probably because of the low sensitivity of measurements in batch culture in microtitre plates). Another factor that should be considered is dosage imbalance as a result of deviation from wild-type protein levels ([24-27]).

**Figure 6:**
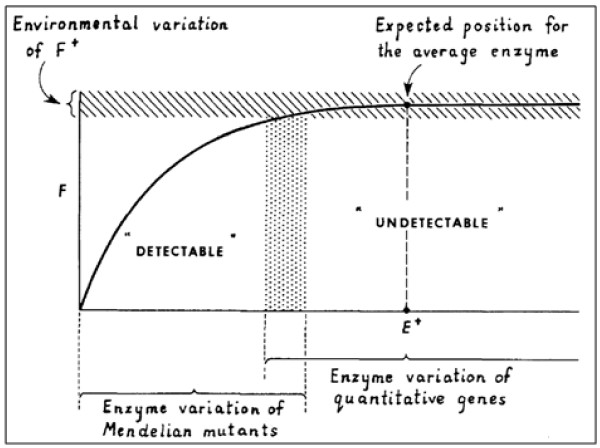
**The classical view of the enzyme - flux relationship (Kacser and Burns, 1981)**. Reproduced with permission (copyright is retained by the Genetics Society of America).

Kacser and Burns [1] have described the relationship between enzyme activity and flux as a monotonic curve that converges to a saturation level (Figure 6). This curve implies that changes in enzyme activity in the 'undetectable range' do not have a major impact on flux, while small changes can be more easily detected within the 'detectable range'. However, this view of dosage *versus *flux is limited for two reasons: (i) Only enzymes are considered, hence increasing an enzyme's level either increases the flux or has no effect on it (monotonic). (ii) It was assumed that enzymes are unlikely to have a mutation that will increase their activity (and that, even if they did, the effect would be in the 'undetectable' range). Indeed, we have not found any enrichment of genes that encode enzymes in our set of HFC genes. Hence, a saturation curve will not explain the phenotypes observed in a genome-wide screening of HI and HP mutants.

In order to explain our results with a dosage *v. *flux curve, we have to extend its applicability to all the cases we observe and we should expect to find a different curve for each protein. For example, the lack of correlation between dosage compensation and high flux control demonstrates that the threshold lies at a different level of activity for each gene or protein. Further, haploproficiency cannot be explained by a curve that converges to a saturation level, as the maximum growth rate of a heterozygous mutant can be no higher than the maximum growth rate of the wild type on such a curve. Therefore, the flux and the control exerted on it by a protein have to be the outcome of multiple factors, each of which can be hypothesized to be a non-linear function of gene (protein) dosage/activity. The activity *v. *flux curve shown in Figure 6 has to be coupled with additional functions that explain the contributions or limitations imposed on the growth rate by the protein - either due to its toxic effects and functional inhibition on growth, or to the imbalances that result from changes in its abundance.

**Figure 7:**
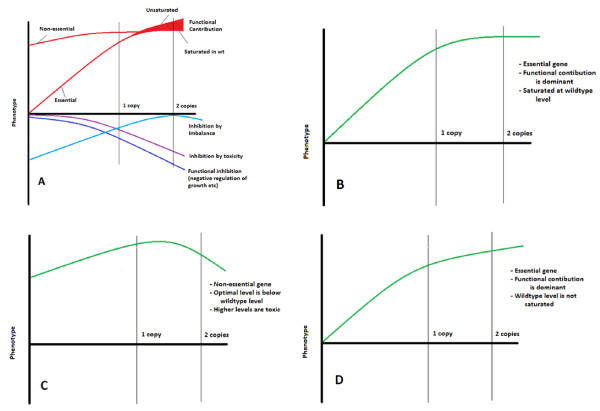
**Phenotype-gene copy number relationships**. **A**. Hypothetical relationship between phenotype and gene copy number. **B-D **Hypothetical examples of superimposed phenotype-gene copy number curves.

In conclusion, we hypothesize that a more realistic representation of the link between protein abundance and flux control can be achieved by a superimposition of curves, as shown in Figure 7A. We have extended the classical view shown on Figure 6 by including four more factors:

1. The possibility of non-zero flux, even if the protein concentration is zero, and the possibility of achieving higher fluxes as the protein concentration is increased to higher levels than in the wild type. The former possibility applies to non-essential genes in a diploid organism, and the latter to proteins that increase the flux when they are over-expressed.

2. Dosage imbalance: Components of the tubulin complex [24] and cell-cycle-related processes [27] have been shown to cause dosage imbalance if their concentration deviates from wild-type levels.

3. Inhibition by toxicity: Over-expression of hundreds of genes has been shown to cause toxicity [22] and [23]; the mechanism of toxicity is not known for most of these genes.

4. Functional inhibition: The function of many proteins is the inhibition or delay of processes that lead to cell growth. The timing and level of expression of genes encoding such proteins is usually fine-tuned by the cells to avoid unwanted inhibition of growth. Our results indicate that cell cycle checkpoint proteins may exert a significant inhibition on growth rate.

Hypothetical examples of such curves are given in Figures 7B-D. It is likely that genes with curves as shown in Figures 7C and 7D constitute a considerable fraction of the genome, rather than being exceptional cases. Revealing the superimposed fitness curve for each gene requires data on growth rates at various gene copy numbers (or protein concentrations), examples of which are starting to appear in the literature (Alcasabas and co-workers (submitted); [28]).

**Figure 8:**
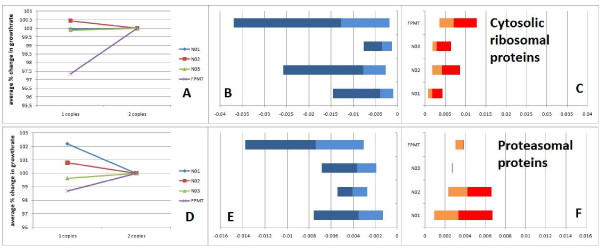
**Phenotype of genes encoding components of protein complexes as a function of copy number is context dependent**. **A**. Cytosolic ribosomal proteins have positive slope in turbidostats **B-C**. The range of significant FCC's for cytosolic ribosomal proteins. **D**. Sign of slope of the phenotype-copy number curve for genes specifying proteasome subunits. **E-F**. Range of significant FCC's for proteasomal proteins.

Two examples of applicability of these principles are shown on Figure 8. Average slope of gene copy number - phenotype curve for genes encoding cytosolic ribosomal proteins is significantly positive in turbidostat selections; however, it is very close to zero in nitrogen-limited competitions. This indicates that cells with reduced numbers of ribosomes can still grow at the population average under nitrogen limitation, probably because competition is not for rapid synthesis of proteins. However, having reduced ribosomal protein gene copies in turbidostats caused growth deficiency, indicating that protein synthesis is rate-determining in turbidostats. Hence, on average, the protein components of cytosolic ribosomes have detectable growth-rate control under the turbidostatic conditions, but not under nitrogen limitation. The range of FCC' values for HFC genes encoding components of cytosolic ribosomes is shown in Figures 8B-C. Similarly, the average slope of the copy number - phenotype curve for genes specifying proteasomal proteins is significantly positive in turbidostat selections. However, the value of the slope is smaller in nitrogen-limited cultures at high growth rates and finally changes its sign to negative at D = 0.2 h^-1 ^and to an even lower value at D = 0.1 h^-1 ^(Figure 8D). Both ribosomal and proteasomal proteins span a large range of negative FCC's while proteasomal proteins span the largest range of positive FCCs', indicating the variation of FCCs' within a protein complex and context dependency of flux control. Hence the dosage vs flux curve for a protein identifies how its function, toxicity, imbalance, abundance, or redundancy reflects on the growth rate.

## Discussion

Haploinsufficiency is the situation where the reduction in the copy number of a gene in a diploid organism from two to one results in a significant loss of fitness. It is assumed that the reduction in copy number results in a corresponding reduction in the concentration of the gene's protein product, such that there is insufficient of that protein present to sustain the wild-type phenotype. The connection of haploinsufficiency to diseases has long been known [29-31] and various hypotheses have been proposed to explain the mechanisms of haploinsufficiency ([26,25]). The kinetics of gene expression in connection to haploinsufficiency has also been studied [32]. Haploinsufficiency was initially identified in gene-by-gene phenotype screenings in humans and other organisms. However, the availability of a genome-wide collection of deletion mutants, as well as tag arrays for quantifying the proportions of individual mutants in a population, has enabled genome-wide screening for phenotypes of *S. cerevisiae *deletion mutants under various conditions ([5,33,4]). Previously, sets of HFC genes have been reported for the following cell populations grown in batch culture: (i) homozygous deletion mutant pool growing in YPD [5]; (ii) both homozygous and heterozygous pools growing on fermentable and non-fermentable carbon sources [34]; and (iii) heterozygous and homozygous pools growing in YPD and minimal medium [35]. Some of these pools have also been screened for haploinsufficiency when treated with drugs and sets of genes with drug sensitivity and resistance have been identified ([36-38]).

Haploproficiency under fast growth rate conditions has not been reported until recently [39] in genome-wide phenotype screenings of *S. cerevisiae*. However, it has been reported that it is possible to select for mutants with 5-50% increase in growth rate (as compared to wild type) under specific nutrient limitations [40]. Some of these evolved strains had transporter gene amplifications, together with other mutations not associated to related physiology. It was later reported that *Kluyveromyces marxianus *[41], allowed to evolve in a mineral medium, can attain higher growth rates than the mother strain. Changes in the morphology of these cells were found to be linked to the increased maximum growth rate, probably because a larger surface area (relative to cell volume) of the elongated mutants allows transport processes to take place more efficiently. See the review by Cakar and co-workers for recent approaches in evolutionary engineering of strains [42].

Although data on the evolution of strains have provided a valuable resource for elucidating the mechanisms of growth rate control, systematic screens of single-gene deletion mutants provide genome-wide profiling of all known genes. While mutants with severe growth defects can be easily detected in such screenings, it has been difficult to detect single-gene deletion mutants which grow faster than the wild type, as the difference in growth rate can be small and below the threshold of detection. This hindered the identification of haploproficient hemizygotes in many studies. We have shown that competition at slow growth rates in nutrient-limited chemostats can be used to identify growth advantages as well as growth deficiencies at high sensitivity [4]. Most of the genes only displayed haploproficiency under a particular nutrient limitation; for example, most genes encoding subunits of the 26S proteasome were HP under nitrogen limitation at D = 0.1 h^-1 ^[4]. The reason for their haploproficiency under these conditions is unknown; however, it is likely to be a positive outcome of reducing the rate of protein turnover when there is competition for nitrogen resources.

In a recent study on *Schizosaccharomyces pombe *[39], 136 heterozygous deletion mutants in competition were shown to have faster growth rates than wild type when grown in batch on a rich medium. The same study also reported 183 haploproficient *S. cerevisiae *genes derived from a re-analysis of previously published data from a competition of heterozygous deletion pools under similar conditions [35]. Screening of a library of hypomorphic alleles of essential genes also revealed a small set of faster-growing mutants with a reduced concentration of essential proteins [43]. In this study, we have identified a large set of haploproficient as well as haploinsufficient hemizygotes using *S. cerevisiae *cultures growing at their maximum growth rates. Although these results are novel, it is not surprising that reducing the level of proteins that act as negative regulators of processes required for rapid growth, or that are toxic as monomers in the cytosol, can have a positive impact on growth rate. Although we might expect such growth-rate-limiting factors to be eliminated by evolution in order to attain yet higher growth rates and thus greater fitness, it is possible that these factors were optimized rather than eliminated, without compromising the basic function of the protein, which may be essential under sub-optimal conditions.

Our HI gene set includes 124 of the 186 HI genes reported by Deutschbauer and co-workers [35] (GR < 0.97) (our set had data on 166 of the 186), while 8 of the 166 are significantly HP in our set. Our HP gene set includes only 21 of the 181 HP genes reported by Deutschbauer and co-workers [35] (GR > 1.03), 178 of which exist in our data set. 46 of these genes are HI in our set, demonstrating that the results from the two experiments vary significantly with respect to the identification of the HP phenotype. There can be a number of reasons for the discrepancy between the two datasets. First, our classification criteria are based on goodness-of-fit rather than the percent change in growth rate - so mutants with small, but significant, changes are included in our HFC set, while mutants with large changes may have been excluded if their readings were noisy. Moreover, the experimental regimes used in the two studies are different since continuous turbidostats were used in this study as opposed to batch shake flasks. Finally, both experiments are expected to have some level of stochasticity in both the competition and sampling steps, and downstream processing of the samples may also introduce noise into the final results.

### Features of genes and proteins are not globally correlated to flux control

Haploinsufficiency can be linked to a number of factors, and previous authors have found evidence for the HI phenotype of hemizygous deletants being the result of either the insufficient concentration of the protein encoded by the single-copy gene or of dosage imbalance [35]. Noise in gene expression has also been proposed as a reason for haploinsufficiency ([32,44]), while Veitia [45] suggested synergistic interactions between transcription factors (TF) as the cause of the haploinsufficiency of TF mutants. Accordingly, we compared our FCC' measurements to data from the literature to determine whether these, or any other factors, had an important impact on FCC' values in rapidly growing yeast.

Haploinsufficiency is often described as a direct consequence of inadequate concentrations of proteins [35], so it can be hypothesized that the proteins with higher abundance will have higher flux control when their gene copy number is reduced - particularly if dosage compensation in hemizygous mutants is rare, as reported by Springer and co-workers [21]. However, we found no significant correlation between protein abundance [46] and high flux control at rapid growth. It was also hypothesized that proteins with noisy expression are more likely to fall below the threshold [31] that will cause irreversible growth defects. We have compared modified flux control coefficients to noise in mRNA expression (DV values from [47]). There was no genome-wide correlation between high growth-rate control and noise in mRNA expression, nor were the rates of mRNA and protein decay or half-lives [48-50] correlated to growth-rate control. In a recent study on haploinsufficiency in humans [51], genetic interaction network features were identified as the best predictors of haploinsufficiency. However, the correlation between the number of genetic interactions of genes and their FCC' value in our turbidostat study were insignificant (rho = -0.049 for positive interactions, -0.055 for negative interactions, -0.015 for synthetic lethal, rho = -0.065 for all physical and genetic interactions of yeast from BioGrid, [52]). We have also demonstrated that, although growth-regulated genes (from our Category 1 experiments) are, themselves, controllers of flux at the fastest growth rates, there is no simple relationship between the change in growth rate due to haploinsufficiency and the fold change in transcript level or protein concentration.

Haploinsufficiency has been linked to the stoichiometry of protein complexes as there is evidence that dosage imbalance has a profound effect on phenotype in some cases ([25,29]). Previous competition experiments on homozygous and heterozygous deletion mutant pools grown in batch on YPD have also shown that genes encoding the components of protein complexes are enriched among the HI genes [35]. Our HFC genes are also enriched in genes encoding components of protein complexes (hypergeometric distribution, p-value = 1.5e^-7 ^for HI genes, p-value = 0.034 for HP genes; the genes assigned to GO Term 'protein complex' were used in the analysis (Saccharomyces Genome Database, SGD, http://www.yeastgenome.org). Moreover, HI genes and their protein products are involved in more genetic or protein-protein interactions than expected by chance (p-value = 8.7e^-7 ^for HI as opposed to p-value = 0.22 for HP, data from Biogrid [52]). Both HI and HP genes are more likely to be essential than the average for the genome (hypergeometric distribution, p-value = 0.0051 for HI genes and p-value = 0.039 for HP genes; list of essential genes downloaded from SGD). These results demonstrate that protein complexes, or proteins that function in interaction with others, are more likely to be involved in high flux control when compared to proteins that act as singletons in the cell.

## Conclusions

Our method has identified many more HI and HP genes than have been reported previously. Our results cover 124 of the 166 HI genes previously identified in YPD [35]. The relatively poor overlap with previous data on HP genes indicates systematic differences between experimental design and data analysis regimes in the two different approaches.

We find that translation and transcription are the bottlenecks for fast growth when the nutrients are not limited. The enrichment of cell cycle genes in the set of genes that are toxic upon over-expression supports our hypothesis that reduction in protein levels increases growth rate as wild-type levels limit the growth rate by 'functional inhibition'. Accumulation of cells in the G1 phase may be an indication of its high flux control, although no direct link exists between the two. Genes involved in the maintenance of genome integrity also show a haploproficient phenotype under conditions of rapid growth. Thus the checks and balances that the yeast cell imposes on itself to ensure that its genome integrity is maintained and that the genome is faithfully replicated and segregated during the cell cycle limit the absolute rate of growth which is compatible with the organism's long-term survival.

Proteins may have different levels of flux control under different growth conditions; their flux control can even be in opposite directions depending on the nature of the competition. Hence, high flux control is context-dependent and is not a direct consequence of any single factor, such as protein abundance or growth-rate regulation of gene expression. It is more likely to be a complex function of protein activity, which can be hypothesised as a superimposition of functions set by multiple factors.

## Methods

### Competition Experiments

A 1 ml aliquot of the heterozygous deletion mutant (BY4743, *MAT**a**/MATα his3Δ1/his3*Δ1 *leu2Δ0/leu2Δ0 lys2Δ0/LYS2 MET15/met15Δ*0 *ura3Δ*0*/ura3*Δ0) pool (obtained from the Yeast Genome Deletion Project library and prepared as described in Additional File 1, Additional Methods) were grown in 100 ml of YPD media overnight at 30°C with shaking at 180 rpm. 10 ml of this preculture was used to inoculate 1L of the appropriate nitrogen-limited or FPM growth media in 2L fermentors. Cultures were grown overnight at 30°C with aeration and stirring at 750 rpm. After 24 hours the fermentors were switched to continuous growth with pH control (set point = 4.5) and either a fixed dilution rate (chemostat) or dilution rate determined by biomass (turbidostats). In turbidostat mode [53], the biomass corresponding to approximately half of the maximum reading from batch phase (corresponding to mid-exponential growth). 20 ml samples were taken from the preculture and overnight batch culture followed by at least four at approximately 24-hour intervals from the continuous cultures. Experiments were made with at least two biological replicates for each fermentation and two technical replicates for each sample. For further details on fermentations including instrumentation and sampling, see the Additional File 1, Additional Methods section.

### DNA extraction, tag amplification, hybridization

Total DNA was isolated from each of the samples using a Promega Wizard Genomic DNA purification kit, according to the protocol provided together with the kit. Universal primers were then used to amplify the up-tag and down-tag sequences with separate PCR reactions as described previously [4]. The PCR products were purified, quantified and hybridized on custom Affymetrix Tag3/4 arrays as described previously ([4,54]).

### Data analysis

Tag3 data were normalised and modelled as described previously [4]. Tag4 data were normalized as described [54] and modelled as described previously [4]. In both cases a relative growth rate and multiple testing corrected P-value (null hypothesis that relative growth rate is equal to 0) were obtained for each strain. The 182 deletion mutants listed in [55] were excluded from further analysis. The relative growth rates were used to calculate the Spearman correlation coefficients between experiments shown in Figure 1B. In Figure 1B the dendrogram showing the relationship between different experiments was constructed using the Euclidean distance between rows (experiments) and complete linkage hierarchical clustering. The functional analysis was made using Gene Ontology [56] as described in the Additional File 1, Additional Methods. All statistical methods were implemented either in R/Bioconductor [57] using RSRuby [http://rubyforge.org/projects/rsruby/] or Matlab [The Mathworks, Inc]. For further details on sample processing and data analysis, see the Additional File 1, Additional Methods section.

### Derivation of modified flux control coefficients

Equation 1 shows the change in abundance of a mutant in a chemostat (or turbidostat) as a function of time:

(1) $$\frac{dX}{dt}=\mu X-DX$$

Where X denotes hemizygote concentration (abundance in terms of number of cells, in arbitrary units) in the chemostat, t denotes time, μ denotes the growth rate of the mutant and D is the dilution rate, which is equal to the average growth rate of the population. After rearranging Equation 1, a linear relationship between the change of log.-transformed hemizygote concentration and change in time can be derived (Eq.2). Thus, linear regression of log.-transformed hemizygote content of the samples with respect to time allows us to calculate the relative difference between the growth rate of the hemizygote and the population average (D)

(2) $$\frac{d\left(lnX\right)}{dt}=\mu -D$$

Each mutant in the heterozygous deletion pool has half the wild-type copy number of one of its genes (reduction from two copies to one), and the change in the gene copy number introduces a deviation from D, so the relative growth rate of a mutant linearly relates to the 'alternative deviation index' (an approximation to the flux control coefficient, or FCC, for large perturbations; [58]) of the gene copy number of the corresponding gene:

(3) $$\begin{array}{ll} DI & =\frac{\partial \mu /D}{\partial copy\;number/copy\;number}\approx \frac{\Delta \mu /D}{\Delta copy\;number/copy\;number}=A\left(\frac{\mu -D}{2-1}\right)\;\\ & =A\frac{d\left(lnX\right)}{dt}\;\\ & \; \end{array}$$

where A is the scaling factor (initial copy number/D).

Since A is a constant that applies to all mutants in the pool, the relative growth rates of the mutants can be used to represent the FCC of the corresponding gene with the reduced copy number. We will call this quantity the "modified FCC" or FCC':

(4) $${\text{FCC}}^{\prime }=\frac{d\left(lnX\right)}{dt}$$

Note that the FCC' value of a gene depends on the local slope of a protein concentration vs fitness curve at the range of interest (Figures 6 and 7 give examples of such curves). For example, the FCC' of an essential gene can be very high in the range of 0 copies to 1 copy (change in flux in heterozygous deletion mutant with respect to the inviable null mutant), or the FCC' of a gene whose protein product is at a concentration higher than needed in the wild-type cell *(i.e. *a level beyond saturation) would be close to zero at proximity of the wild-type level. In this study, the relative growth rate (or FCC') gives the negative average slope of the protein concentration vs fitness curve between 1 copy and 2 copies of each gene, *i.e. *our HI genes with negative FCC's indicate a positive flux control by the gene when its copy number is increased from 1 to 2, and similarly our HP genes indicate a negative flux control. These principles apply both for gene copy number and for protein abundance, based on the assumption that diploid cells with only one copy of a gene always have a lower concentration of its protein product than the cells with two copies.

### Cell cycle analysis

Cell cycle profiles were determined using the method described in Haase and co-workers [59]. Briefly, cultures were grown to exponential phase before fixation in 70% v/v ethanol, followed by RNase A and protease treatment. Washed samples were then mixed with 1 μM Sytox Green and analysed for DNA content in a Beckman Coulter CyanADP flow cytometer. 20,000 events were captured for each sample with three biological replicates (separate cultures) for each strain. Two peaks were fit to the count histogram of DNA content using the Dean-Jett-Fox method [60] and the percentage of the total cells under the G1 peak was recorded for each sample.

## Authors' contributions

PP, AG, JW, BH and NZ designed and built the experimental setup; and carried out the experimental work. PP, AG and SGO performed the data analysis. SGO and DBK designed the study; SGO supervised the work. PP, AG and SGO wrote the manuscript. All authors read and approved the manuscript.

## Supplementary Material

Additional file 1 **Additional Text and Methods**. Additional text on results and details on methods are provided. Click here for file

Additional file 2 **Figure S1. Histogram of relative growth rates (FCC') of HFC genes and all genes in turbidostats**. HI: Haploinsufficient, HP: Haploproficient (FDR < 0.05 is the threshold for significant HI and HP genes). FCC's of 5713 genes (genome), 1932 HI genes and 796 HP genes were binned into 50 intervals each. Click here for file

Additional file 3 **Table S1: Relative growth rates from the experiments**. Abbreviations: C, N, P: Carbon-, Nitrogen- or Phosphorus-limited F1 media 01, 02, 03: Dilution rates in chemostats of 0.1, 0.2 or 0.3 h^-1^, respectively Turb: Turbidostat FPM: Footprinting media Columns: 1: Systematic name of the ORF, one copy of which has been deleted in the hemizygous diploids All columns denoted as .FCC': Relative growth rate (modified control coefficient) of the mutant in the corresponding experiment. All columns denoted as .P: P-value for rejecting the hypothesis that GR ≠ 0. All columns denoted as .FDR: False discovery rate at the specified P-value. Click here for file

Additional file 4 **Table S2: GO analysis of the growth rates**. Each experiment is given on a separate spreadsheet. Abbreviations: C, N, P: Carbon-, Nitrogen- or Phosphorus-limited F1 media 01, 02, 03: Dilution rates in chemostats of 0.1, 0.2 or 0.3 h^-1^, respectively Turb: Turbidostat FPM: Footprinting media Columns: 1: Internal database row number. 2: GO term ID 3: Name of the GO term 4: Class of the GO term (MF: Molecular Function, BP: Biological Process, CC: Cellular Compartment) 5: Number of genes annotated to the GO term 6: Coeff: Logistic regression coefficient β of the GO term. Positive values indicate enrichment of a GO term among the haploproficient genes, negative values indicate enrichment among the haploinsufficient genes. 7: Odd.ratio: Log odds ratio of the GO term. 8: P-value: P-value for rejecting the null hypothesis that β≠0 9: FDR: False Discovery Rate at specified P-value. Click here for file

Additional file 5 **Figure S2. Fraction of HFC genes related to selected organelles, protein complexes, and cellular processes**. Only genes showing an HFC phenotype in turbidostat culture are considered. The key on the top left gives the colour code used in the chart: Dark blue gives the percent of significantly HI (FDR < 0.05) and dark red gives the percent of significantly HP (FDR < 0.05). Click here for file

Additional file 6 **Table S3: Haploproficient genes involved in the maintenance of genome integrity**. Columns: 1. ORF name 2. Gene name 3. Gene description 4. Relative growth rates (FCC') 5. FDR: False Discovery Rate. Click here for file
