# Supplementary material for: The genetic control of growth rate: a systems biology study in yeast
Source: BMC Syst Biol. 2012 Jan 13;6:4. doi: 10.1186/1752-0509-6-4 (PMC3398284; doi:10.1186/1752-0509-6-4)
Supplement: Additional file 1 — Additional Text and Methods. Additional text on results and details on methods are provided. [file 1752-0509-6-4-S1.DOC]

**Additional Text (Pir *et al.*, *BMC Systems Biology*, 2012)**

**Chemostat and Turbidostat Cultures**

Chemostats are continuous cultures grown in fermentors with control systems, which are widely used for research in microbial physiology [1]. Parameters, like temperature, pH, and concentration of dissolved gases in the growth medium, can be monitored and controlled. Fresh medium is supplied to the culture at a constant flow rate (F), and removed from the fermentor at the same rate, thus maintaining a constant culture volume (V). At steady state, the growth rate of the culture equals to the dilution rate, D = F/V, and the biomass concentration and other free parameters stabilise at levels depending on the dynamics of the fermentation. Hence, chemostats allow continuous exponential growth to occur for many generations under constant physicochemical conditions. Crucially, chemostat culture allows the experimenter to control the growth rate (flux) at any value below a maximum value termed the maximum specific growth rate (μ_max_). This contrasts with batch cultures, where biomass concentration and the environmental conditions (in terms of pH, nutrient concentration etc.) change significantly during the (limited) time course of the fermentation, and the experimenter has no control over growth rate (flux).

The factor that determines the growth rate of a cell population in a chemostat is dilution rate, *i.e.* the rate of supply of the limiting nutrient. In a chemostat operating at a low dilution rate, the limiting nutrient is present at very low concentrations at the steady state. Therefore, most of the nutrient is converted into cells and the biomass concentration is high (close to the cell concentration of an equivalent batch culture at late exponential phase). As the dilution rate is increased, the availability of the limiting nutrient increases; however, the rate of removal of cells from the growth vessel is also higher and so the biomass concentration falls. At values of D _˜_ μ_max_ , nutrient limitation entirely disappears as there are too few cells in the culture to utilize the available nutrients. Those cells remaining in the culture vessel grow at their maximum growth rate since even the limiting nutrient is present in excess. Finally, at D > μ_max_ , a steady state can no longer be maintained and the number of cells in the fermentors starts to fall since the rate at which new cells are produced is insufficient to overcome dilution by the addition of fresh medium, which leads to the washout of the cells from the growth vessel.

## Turbidostats, are continuous cultures in which the cells, rather than the experimenter, control the growth rate [2]. A turbidostat employs a positive feedback control system that senses, and responds to, the biomass concentration in the fermentor. If the culture is growing faster than the rate imposed by the current dilution rate, then the biomass concentration will rise as a result of the positive difference between the biomass production rate and the rate of removal of cells from the culture vessel. In such circumstances, a control loop is activated that increases the dilution rate by increasing the speed at which a pump introduces fresh medium into the culture. This continues until the biomass concentration is decreased to some set point (which, in our experiments, represents the biomass concentration of a mid-exponential phase batch culture growing in the same medium under the same conditions). Contrariwise, if the culture is growing slower than the current dilution rate, the biomass concentration will fall to below the set point, and the control loop acts to decrease the rate at which fresh medium is pumped into the growth vessel. Hence the turbidostat equilibrates at a dilution rate equal to the actual growth rate of the culture and the cell concentration stays constant. As our set point is equivalent to the biomass concentration of a mid-exponential phase batch culture, the turbidostat equilibrates at the maximum specific growth rate of the yeast strain used. However, if the culture is a pool of heterozygous deletion mutants of *S. cerevisiae*, and if the different mutants in the pool can have different maximum growth rates, those mutants that can achieve a μ_max_ greater than the population average will increase in the population over time, while those with a μ_max_ less than population average will decrease in the population. Thus turbidostats, just like chemostats, represent a sensitive way of identifying haploinsufficient and haploproficient phenotypes, with the difference that (in a turbidostat) haploproficient mutants must be capable of growing at a rate greater than the previously recorded μ_max_.

**Genome-wide Distribution of FCC’**

Additional Figure 1 shows that the number of genes showing an HI phenotype is *ca.* twice that of the genes showing an HP phenotype (henceforth, for convenience, we shall refer to them as HI and HP genes). This demonstrates that more mutants had a slower growth rate than the population average than had a faster growth rate. One reason for this imbalance could be that the slow-growing mutants are decreasing in number in the population and so their contribution to the average growth rate of the population is decreasing over time. We limited our competitions to 30 generations to avoid any possible build-up of secondary mutations; however, if the competition were carried out for many generations, a measurable time-dependent shift in the average growth rate of the population should have been observed as a result of slow-growing strains being washed out of the turbidostat and fast-growing mutants dominating (and increasing) the average growth rate. In fact, there was no detectable shift in the dilution rate over time and our FCC’ calculations are based on the assumption that the average population growth rate was constant throughout the study.

The relative growth rate of the standard strain *HO*/*hoΔ* which we previously showed to differ from the wild-type fitness by ≤ 3% [3], changed by -1.7% in our competition experiments. The significance of this change was close to the threshold (FCC’_HO_ = -0.0056, FDR = 0.04). This demonstrates that the population average in our competition can safely be taken as the wild-type growth rate, although there may be a small bias towards haploinsufficiency as a result of a shift in the population average explained above.

**Reproducibility of Results**

The maximum positive Spearman correlation coefficient for FCC’ values obtained under two different growth conditions was 0.59 (for C- and N-limited chemostats operating at D = 0.1 h^-1^, Figure 1 in the main article). We compared this to the correlation coefficients obtained between biological replicates of the same growth conditions and found this to vary between 0.70 and 0.93 (p ≈ 0). This indicates that, although the stochastic nature of competition in fermentors and the downstream sample processing introduce noise, the differences between conditions are still significant.

It is possible that interactions between mutants in the pool represent another source of variation in competition experiments. To test this possibility, we built a heterozygous deletion mutant pool with 200 mutants selected from the set of HFC genes from turbidostats. Our detection method for mutants is hybridization-based; hence, the number of competing mutants has to be relatively large in the pool to avoid saturation of probes on the Tag3 arrays. Competing only small number of mutants, *i.e.*, only a reference mutant and a test mutant would be infeasible for this reason, in addition to being costly and labour intensive. We have competed the 200 mutants in the `small pool` under the same conditions as the genome-wide pool of mutants. The mutants which were HI in the genome-wide pool were also HI in the small pool, and the mutants which were HP in the genome-wide pool were also HP in the small pool. This indicates that our results from turbidostats are reproducible in a culture with a different combination of mutants (results not shown). However, most mutants found to be HI or HP in turbidostats do not exhibit any detectible change in their growth rate with respect to wild type when they are grown on their own in FPM in microtitre plates, as expected (results not shown).

**Additional Methods**

**Mutant Pool**

Strains from the heterozygous deletion mutant library (BY4743, MAT**a**/MATα his3Δ1/his3Δ1 leu2Δ0/leu2Δ0 lys2Δ0/LYS2 MET15/met15Δ0 ura3Δ0/ura3Δ0) from the Yeast Genome Deletion Project library (<http://www.sequence.stanford.edu/group/yeast_deletion_project/deletions3.html>) were grown in 98-well plates in YPD for two days at 30^o^C and equal amounts of each culture were mixed to prepare plate pools. Equal amounts of the well-mixed plate pools were mixed to prepare the genomic pool of heterozygous deletion mutants. Aliquots (1ml) of the pool were then stored at -80^o^C.(A small pool composed of 200 selected mutants were prepared using the same method.)

**Preculture**

1ml aliquot of heterozygous deletion pool was grown in 100ml of YPD for 24 hours in an orbital shaker kept at 30^o^C and 180rpm. The culture was sampled and the cell pellet from two 20 ml samples was stored at 80^o^C until further processing.

**Fermentations**

Ten ml of the preculture was used to inoculate 1L of FPM or nitrogen-limited F1 medium in the sterilized fermentor vessels. The composition of FPM [4] and nitrogen-limited F1 [3] are as follows:

FPM: Yeast Nitrogen Base (1.7g/l), ammonium sulfate (5g/l), arginine (0.174g/l), aspartate (0.171g/l), glutamate (0.169g/l), histidine (0.155g/l), leucine (0.131g/l), lysine(0.183g/l), methionine (0.149g/l), serine (0.105g/l), threonine (0.119g/l), tryptophan (0.204g/l), valine (0.117g/l), citrate (0.210g/l), fumarate (0.160g/l), malate (0.134g/l), pyruvate(0.110g/l), succinate (0.270g/l), cytosine (0.111g/l), uracil (0.112g/l), glucose (20g/l)

N-lim F1: KH_2_PO_4_ (2g/l), MgSO_4_·7H_2_0 (0.55g/l), NaCl (0.1g/l), CaCl_2_·2H_2_O (0.09g/l), Uracil (0.02g/l), L-Histidine (0.02g/l), L-Leucine (0.1g/l), ZnSO_4_·7H_2_O (0.7 × 10^-4^g/l), CuSO_4_·5H_2_O (0.1 × 10^-4^g/l), H_3_BO_3_ (0.1 × 10^-4^g/l), KI (0.1 × 10^-4^g/l), FeCl_3_·6H_2_O (0.5 × 10^-4^g/l), myo-inositol (0.12g/l), thiamine/HCl (0.014g/l), pyridoxine (0.004g/l), Ca-pantothenate (0.004g/l), biotin (0.0003g/l), ammonium sulfate (0.46g/l) and glucose (20g/l)

The cultures were first grown in batch in fermentors kept at 30^o^C with 1L/min aeration and 750rpm stirring for 24 hours. During batch culture, no pH control was made and within 24 hours the pH dropped from 4.5 to approximately 3. After 24 hours, the fermentors were sampled and switched to continuous mode (either fixed dilution rate or turbidostat mode), pH control was also started at this point and kept at 4.5 until the end of the fermentation. The fermentors were sampled every 24 hours (except for the nitrogen limited D=0.2h^-1^ experiment, see below for details) and the cell pellets from two 20-40ml samples were kept at 80^o^C until further processing. Fermentors were run in continuous mode until 4 time points were collected in addition to the first time point sample from the preculture and second time point sample from the initial batch growth. Experiments were made with at least two biological replicates for each fermentation and two technical replicates for each sample.

**Chemostats**

Chemostat experiments were carried out under fixed dilution rate after batch growth in YPD as described above. The carbon, nitrogen and phosphorus-limited D=0.1h^-1^ experiments were sampled at 0, 5, 21 and 32 generations. Nitrogen D=0.2h^-1^ experiments were sampled at 0, 7.6, 20.8 and 29.1 generations. FPM D=0.3h^-1^ experiments were sampled at 0, 10.4, 20.8, 31.1 generations. Number of generations were calculated using the doubling time formula (ln(2)/D).

**Turbidostats**

Turbidostat experiments were carried out identically to the chemostats, except for dilution rate control [5]. Instead of a fixed dilution rate, a target biomass content was specified such that biomass was maintained at that seen during mid-exponential growth. Turbidity (biomass content) was measured using Aber Instruments turbidity probes [6] and Aber Instruments Biomass Monitor/Applikon DI 1030 Biocontroller system was used for monitoring and controlling. The mid-exponential turbidity reading from the batch growth (approximately half of the maximum) was set as the target biomass content for continuous mode, and the input medium flow was controlled by a pump connected to the control system to keep the biomass content at this set point. The output pump was operated at its maximum rate to pump out excess culture, so the volume of the fermentor was kept at 1L at all times. The maximum growth rate reached in FPM was approximately 0.32h^-1^ and cultures were sampled at 0, 11.0, 22.2, 33.2 generations. Same methodology was followed for cultivating and sampling the small pool of 200 mutants.

**DNA Isolation and PCR**

Approximately 1/10 of the cell pellets were used for DNA isolation, using Promega Wizard Genomic DNA purification kit, according to the protocol provided together with the kit. Isolated DNA was visualized on 1% agarose gel and quantified via NanoDrop. 200ng of the DNA from each sample was used to prepare the PCR reaction aliquot, by adding 192 μL of Invitrogen Platinum PCR Supermix and 100ng of up-tag or down-tag primers, and made up-to 200 μL using water. PCR reactions for amplifying up-tags and down-tags were carried out separately using the following biotinylated universal primers synthesized by MWG/Operon:

B-U1 5’-GATGTCCACGAGGTCTCT-3’

B-U2 comp 5’-GTCGACCTGCAGCGTACG-3’

B-D1 5’-CGGTGTCGGTCTCGTAG-3’

B-D2 comp 5’-CGAGCTCGAATTCATCG-3’

In each case the 200 μL PCR mix was divided into 50 μL aliquots and amplified using the following PCR program: 95^o^C 5 min., (94^o^C 20 sec., 56^o^C 20 sec., 72^o^C 30 sec.) x 35 cycles, 72 ^o^C 5min.

5 μL of the 200 μL re-combined PCR products were visualized on 2% agarose gel, purified and concentrated to 40 μL using either Microcon YM (Cat No. 42408) or Amicron Ultracell-10K (Cat No. UFC501096) filters, by loading the product to the filters, adding 300μL of water and centrifuging for 15min at 13000g for three times and then eluting the product to a fresh eppendorf tube for 3min at 1000g. 1 μL of the cleaned PCR product was visualized and quantified on 2% agarose gels with respect to PCR Marker (New England Biolabs, N3234L).

**Hybridization**

500ng of the up/down-tag PCR product was used to prepare the hybridization mixes. Hybridization, array washing and scanning was carried out as described previously [7],[8] using custom arrays Tag3 microarrays (Affymetrix) for the carbon, phosphorus and nitrogen D=0.1h^-1^ and D=0.2h^-1^ and the FPM turbidostat samples and Tag4 (Affymetrix, GenFlex 16K v2) for all others using a Genechip 640 hybridization oven, Genechip 450 fluidics wash-station and Genechip 7G scanner.

**Cell cycle analysis**

Cell cycle profiles were determined using the method described in Haase and wo-corkers*.* [9]. Briefly, 100ul of strain preculture was cultured in 10ml of FPM and monitored until it reached exponential phase (0.2 – 0.5 OD_600_). Cells from 1ml of culture were then fixed in 70% v/v ethanol, followed by treatment with RNase A for 2-12 hours at 37^o^C (2mg/ml RNase A in 50mM Tris pH 8.0 15 mM NaCl) and protease solution for 15-20 minutes at 37^o^C (5mg/ml pepsin, 4.5 μl/ml concentrated HCl). Washed samples were then mixed with 1μM Sytox Green in 50mM Tris pH 7.5 and analysed in a Beckman Coulter CyanADP flow cytometer. 20,000 events were captured for each sample with three biological replicates (separate cultures) for each strain. Two peaks were fit to the count histogram of DNA content using the Dean-Jett-Fox method [10] and the percentage of the total cells under the G_1_ peak was recorded for each sample.

**Statistical Methods**

Tag3 data were analysed as described previously [11]. Tag4 data were normalized as described [8] and modeled as described previously [11]. The relative growth rates for each strain were then used to select the top 500 haploproficient (positive relative growth rate) and haploinsufficient (negative relative growth rate) strains for each experiment used to construct the Venn diagrams in Figures 2A-D and to calculate the Spearman correlation coefficients between experiments used to construct Fig.2E. In Fig.2E the dendrogram showing the relationship between different experiments was constructed using the Euclidean distance between rows (experiments) and complete linkage hierarchical clustering.

Functional analysis using GO and KEGG annotations was performed using a logistic regression technique similar to LRpath [12]. The relative growth rates from all strains detected in an experiment were encoded into a vector used as the explanatory variable (*x*). Then for each GO term or KEGG pathway (functional category or gene set), membership of the set was encoded into another vector used as the dependent variable such that a value of 1 indicates membership and 0 otherwise. The two vectors were then used to fit a logistic model (shown in Equation 1 where π is the proportion of gene belonging to the set) using maximum likelihood estimation.


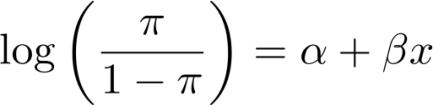
 Equation 1

Parameters α and β are both estimated from the data. The slope of the regression (β) corresponds to the change in the log odds of a gene belonging to the gene set given a unit increase in *x* (the relative growth rate), such that a positive value means that membership of the set is associated with higher relative growth rate (haploproficiency) and a negative value means that membership of the set is associated with lower relative growth rate (haploinsufficiency). The significance of the slope is assessed by calculating the *p*-value for the null hypothesis that β=0, based on the maximum likelihood parameter estimates. This significance is then corrected to control the false discovery rate (FDR) using the method of Benjamini & Hochberg [13]. The same technique was used to find terms where changes in relative growth rate were associated with changes in the overall pool growth rate (Fig.3). In this case the slope of a linear regression of relative growth rate against overall pool growth rate in nitrogen-limitation was used instead of the relative growth rate.

All statistical methods were implemented either in R/Bioconductor [14] using RSRuby [<http://rubyforge.org/projects/rsruby/>] or Matlab [The Mathworks, Inc].

**Additional Figure Legends**

**Figure S1. Histogram of relative growth rates (FCC’) of HFC genes and all genes in turbidostats.** HI: Haploinsufficient, HP: Haploproficient (FDR < 0.05 is the threshold for significant HI and HP genes). FCC’s of 5713 genes (genome), 1932 HI genes and 796 HP genes were binned into 50 intervals each.

**Figure S2. Fraction of HFC genes related to selected organelles, protein complexes, and cellular processes**. Only genes showing an HFC phenotype in turbidostat culture are considered. The key on the top left gives the colour code used in the chart: Dark blue gives the percent of significantly HI (FDR < 0.05) and dark red gives the percent of significantly HP (FDR <0.05) genes in a category. Percent of insignificant HI and HP readings are also given to complement the pie charts. The `Genome` chart just below the key chart gives the genome-wide percentage of HI and HP genes. 34% of the genome is significantly HI while only 13% of the genome is significantly HP.

**Additional References**

1. Bull AT: **The renaissance of continuous culture in the post-genomics age.** *J Ind Microbiol Biotechnol* 2010, **37:**993-1021.

2. Pirt SJ: *Principles of microbe and cell cultivation.* Oxford: Blackwell Scientific; 1975.

3. Baganz F, Hayes A, Marren D, Gardner DC, Oliver SG: **Suitability of replacement markers for functional analysis studies in Saccharomyces cerevisiae.** *Yeast* 1997, **13:**1563-1573.

4. Allen J, Davey HM, Broadhurst D, Heald JK, Rowland JJ, Oliver SG, Kell DB: **High-throughput classification of yeast mutants for functional genomics using metabolic footprinting.** *Nat Biotechnol* 2003, **21:**692-696.

5. Davey HM, Davey CL, Woodward AM, Edmonds AN, Lee AW, Kell DB: **Oscillatory, stochastic and chaotic growth rate fluctuations in permittistatically controlled yeast cultures.** *Biosystems* 1996, **39:**43-61.

6. Kell D, Todd RW: **Dielectric estimation of microbial biomass using the Aber Instruments Biomass Monitor.** *Trends in Biotechnology* 1998, **16:**149-150.

7. Winzeler EA, Shoemaker DD, Astromoff A, Liang H, Anderson K, Andre B, Bangham R, Benito R, Boeke JD, Bussey H, et al: **Functional characterization of the S. cerevisiae genome by gene deletion and parallel analysis.** *Science* 1999, **285:**901-906.

8. Pierce SE, Davis RW, Nislow C, Giaever G: **Genome-wide analysis of barcoded Saccharomyces cerevisiae gene-deletion mutants in pooled cultures.** *Nat Protocols* 2007, **2:**2958-2974.

9. Haase SB, Reed SI: **Improved flow cytometric analysis of the budding yeast cell cycle.** *Cell Cycle* 2002, **1:**132-136.

10. Fox MH: **A model for the computer analysis of synchronous DNA distributions obtained by flow cytometry.** *Cytometry* 1980, **1:**71-77.

11. Delneri D, Hoyle DC, Gkargkas K, Cross EJ, Rash B, Zeef L, Leong HS, Davey HM, Hayes A, Kell DB, et al: **Identification and characterization of high-flux-control genes of yeast through competition analyses in continuous cultures.** *Nat Genet* 2008, **40:**113-117.

12. Sartor MA, Leikauf GD, Medvedovic M: **LRpath: a logistic regression approach for identifying enriched biological groups in gene expression data.** *Bioinformatics* 2009, **25:**211-217.

13. Benjamini Y, Hochberg Y: **Controlling the False Discovery Rate: A Practical and Powerful Approach to Multiple Testing.** *Journal of the Royal Statistical Society Series B (Methodological)* 1995, **57:**289-300.

14. Gentleman RC, Carey VJ, Bates DM, Bolstad B, Dettling M, Dudoit S, Ellis B, Gautier L, Ge Y, Gentry J, et al: **Bioconductor: open software development for computational biology and bioinformatics.** *Genome Biol* 2004, **5:**R80.
