# Supplementary figures and images for: The genetic control of growth rate: a systems biology study in yeast
Source: BMC Syst Biol. 2012 Jan 13;6:4. doi: 10.1186/1752-0509-6-4 (PMC3398284; doi:10.1186/1752-0509-6-4)

## Slide 1
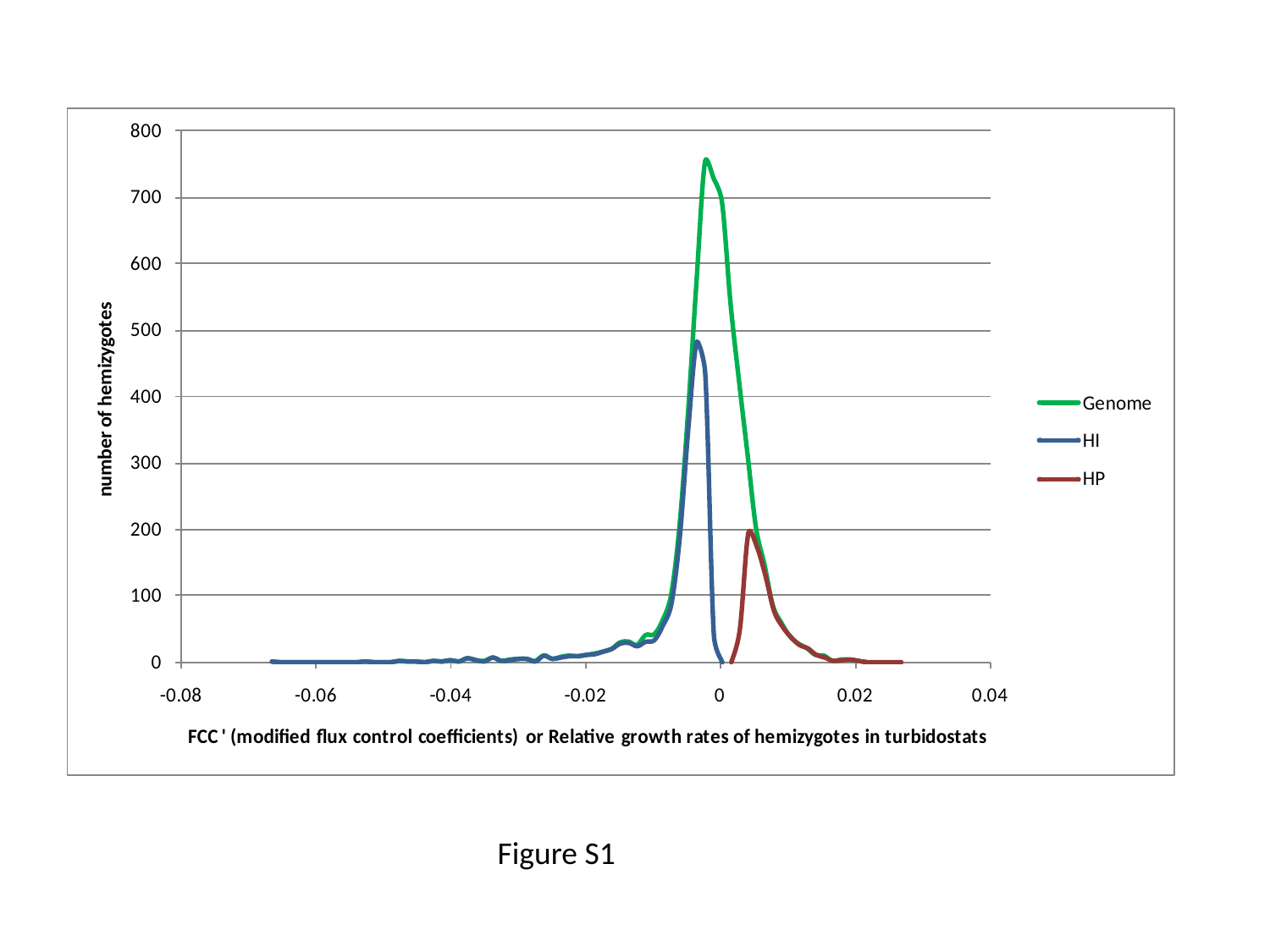

#
Figure S1

Supplement: Additional file 2 — Figure S1. Histogram of relative growth rates (FCC') of HFC genes and all genes in turbidostats. HI: Haploinsufficient, HP: Haploproficient (FDR < 0.05 is the threshold for significant HI and HP genes). FCC's of 5713 genes (genome), 1932 HI genes and 796 HP genes were binned into 50 intervals each. [file 1752-0509-6-4-S2.PPT]

## Slide 1
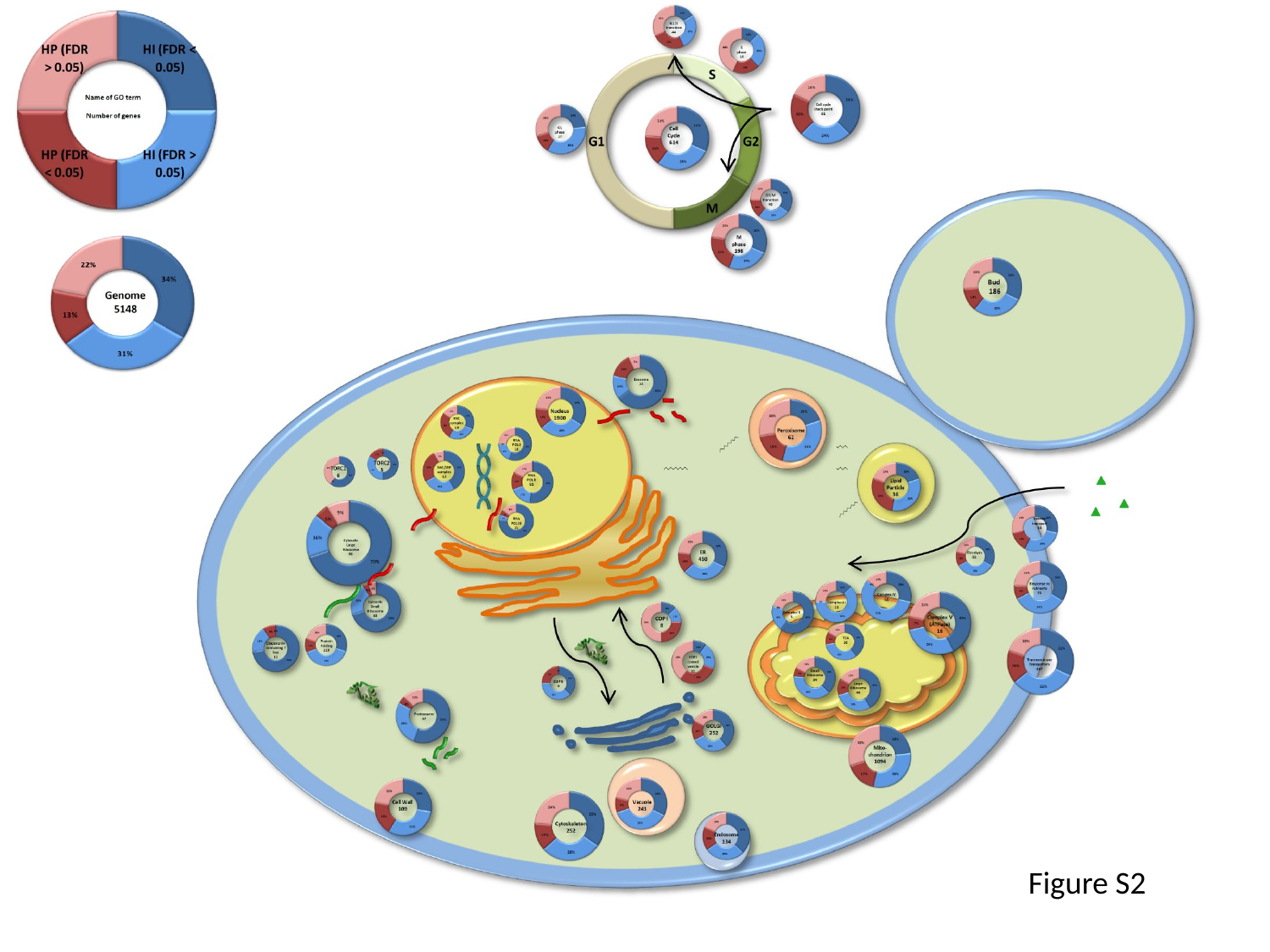

Figure S2

Supplement: Additional file 5 — Figure S2. Fraction of HFC genes related to selected organelles, protein complexes, and cellular processes. Only genes showing an HFC phenotype in turbidostat culture are considered. The key on the top left gives the colour code used in the chart: Dark blue gives the percent of significantly HI (FDR < 0.05) and dark red gives the percent of significantly HP (FDR < 0.05). [file 1752-0509-6-4-S5.PPT]
